# CHARGE syndrome

**DOI:** 10.1186/1750-1172-1-34

**Published:** 2006-09-07

**Authors:** Kim D Blake, Chitra Prasad

**Affiliations:** 1 Department of Pediatrics, IWK Health Centre, Dalhousie University, Canada; 2 Department of Pediatrics, London Health Sciences Center, University of Western Ontario, Canada

## Abstract

CHARGE syndrome was initially defined as a non-random association of anomalies (Coloboma, Heart defect, Atresia choanae, Retarded growth and development, Genital hypoplasia, Ear anomalies/deafness). In 1998, an expert group defined the major (the classical 4C's: Choanal atresia, Coloboma, Characteristic ears and Cranial nerve anomalies) and minor criteria of CHARGE syndrome. Individuals with all four major characteristics or three major and three minor characteristics are highly likely to have CHARGE syndrome. However, there have been individuals genetically identified with CHARGE syndrome without the classical choanal atresia and coloboma. The reported incidence of CHARGE syndrome ranges from 0.1–1.2/10,000 and depends on professional recognition. Coloboma mainly affects the retina. Major and minor congenital heart defects (the commonest cyanotic heart defect is tetralogy of Fallot) occur in 75–80% of patients. Choanal atresia may be membranous or bony; bilateral or unilateral. Mental retardation is variable with intelligence quotients (IQ) ranging from normal to profound retardation. Under-development of the external genitalia is a common finding in males but it is less apparent in females. Ear abnormalities include a classical finding of unusually shaped ears and hearing loss (conductive and/or nerve deafness that ranges from mild to severe deafness). Multiple cranial nerve dysfunctions are common. A behavioral phenotype for CHARGE syndrome is emerging. Mutations in the *CHD7 *gene (member of the chromodomain helicase DNA protein family) are detected in over 75% of patients with CHARGE syndrome. Children with CHARGE syndrome require intensive medical management as well as numerous surgical interventions. They also need multidisciplinary follow up. Some of the hidden issues of CHARGE syndrome are often forgotten, one being the feeding adaptation of these children, which needs an early aggressive approach from a feeding team. As the child develops, challenging behaviors become more common and require adaptation of educational and therapeutic services, including behavioral and pharmacological interventions.

## Disease name/synonyms

CHARGE (Coloboma, Heart defect, Atresia choanae, Retarded growth and development, Genital hypoplasia, Ear anomalies/deafness) syndrome;

CHARGE association;

Hall-Hittner syndrome.

## Definition

The CHARGE association was first described in 1979 by Hall *et al*., in 17 children with multiple congenital anomalies who were ascertained by choanal atresia [1]. In the same year, Hittner reported this syndrome in 10 children with ocular colobomas and multiple congenital anomalies [2], hence the syndrome is also called Hall-Hittner syndrome [3]. Pagon *et al*., in 1981 first coined the acronym CHARGE association [4] (**C**oloboma, **H**eart defect, **A**tresia choanae, **R**etarded growth and development, **G**enital hypoplasia, **E**ar anomalies/deafness) as a non-random association of anomalies occurring together more frequently than one would expect on the basis of chance. The original diagnostic criteria required the presence of four out of six of the CHARGE characteristics. Over the past 15 years the specificity of this pattern of malformations has reached the level that many clinicians now consider it to be a recognizable CHARGE syndrome [1].

## Diagnostic criteria

With increasing experience, it has become clear that the CHARGE association criteria originally proposed by Pagon *et al*. [4], needed further refinement. An expert group of geneticists and developmental pediatricians defined the major and minor criteria of CHARGE syndrome in 1998 [5]. Major criteria are those findings that occur commonly in CHARGE syndrome but are relatively rare in other conditions. The minor criteria occur less frequently or are less specific to CHARGE syndrome (Table 1, first two columns).

**Table 1:** Diagnostic criteria for CHARGE syndrome

Features of CHARGE syndrome		Later childhood/adolescent issues*
Major 4C's **		

Ocular coloboma	**C**oloboma -of iris, retina, choroid, disc; microphthalmia	Photophobia; retinal detachment Corneal abrasions
Choanal atresia/stenosis	**C**hoanal atresia (or Cleft palate) – unilateral/bilateral, membranous/bony, stenosis/atresia	Facial growth problems, recurrent closure and resurgeries, unilateral nasal discharge
Cranial nerve anomalies	**C**ranial nerve dysfunction – Olfactory tract anomalies – Facial palsy (unilateral or bilateral), Sensorineural deafness, Velopharyngeal incoordination – swallowing problems	Feeding/swallowing problems; gastroesophageal reflux; hiatus hernia
Characteristic ear anomalies	**C**haracteristic ear abnormalities – External ear (lop or cup shaped),, Middle ear (ossicular malformations, chronic serous otitis), mixed deafness, semicircular canal +/-cochlear defects	Progressive hearing loss; chronic middle ear infections; vestibular problems affecting balance and/or motor skills.

**Minor**		

Cardiovascular malformations	Cardiovascular malformations – All types: especially conotruncal defects (*e.g. *Tetralogy of Fallot), AV canal defects, and aortic arch anomalies	Arrhythmias; angina; further cardiac surgeries
Genital hypoplasia	Genital hypoplasia – Males: micropenis, cryptorchidism; Females: hypoplastic labia, Both: Delayed incomplete pubertal development	Pubertal delay, hormone replacement; fertility (unsure); hypogonadotrophic hypogonadism, osteoporosis
Cleft lip/palate	Orofacial cleft – Cleft lip and/or palate	Cosmetic concerns; self-image
Tracheoesophageal fistula	Tracheoesophageal fistula – Tracheoesophageal defects of all types	Reflux esophagitis; feeding/swallowing problems
Distinctive CHARGE facies	Characteristic face – sloping forehead, flattened tip of nose	Cosmetic concerns; self-image
Growth deficiency	Growth deficiencies – Short stature Borderline growth hormone (GH) stimulation tests	Growth hormone (GH) replacement Obesity
Developmental delay	Developmental delay – Delayed motor milestones, language delay, mental retardation	Educational, behavioural, social adjustment issues; Autistic-like problems; Obsessive compulsive disorders; Attention Deficit Hyperactivity Disorder (ADHD)

Occasional***		

Renal anomalies	Duplex system, Vesicoureteric reflux	Renal failure
Spinal anomalies	Scoliosis; Osteoporosis	Scoliosis
Hand anomalies	Fifth finger clinodactyly, camptodactyly and cutaneous syndactyly	Fine motor problems; cosmetic concern
Neck/shoulder anomalies	Sloping, Sprengel's deformity, kyphosis	Self-image concern

* A premature aging phenomenom may exist in the older population, however, further research studies are required.

** Absent olfactory pathways and absent/abnormal semicircular canals are cardinal radiological findings for CHARGE syndrome.

***The occasional findings are more prevalent than originally predicted [8–9]

A diagnosis of CHARGE syndrome should be considered in any neonate with coloboma, choanal atresia, asymmetric facial palsy or classical CHARGE ears in combination with other specific congenital anomalies [6] (Table 1). Individuals with all four major characteristics (the classical **4C**'s: **C**hoanal atresia, **C**oloboma, **C**haracteristic ears and **C**ranial nerve anomalies) or three major and three minor characteristics are highly likely to have CHARGE syndrome [5]. In some children, the presence of a cleft lip and palate can substitute for choanal atresia, since the two defects rarely occur together. Some of the CHARGE features are difficult to detect in the neonatal period; therefore, the diagnosis needs to be considered in any infant with one or two major criteria and several minor characteristics. CHARGE syndrome has also occurred in an individual with no coloboma or choanal atresia [7].

## Epidemiology

The true incidence of CHARGE syndrome is not known, with estimates ranging from 0.1–1.2/10,000 live births. A national surveillance study of CHARGE syndrome patients has been conducted through the Canadian Pediatric Surveillance Programme (CPSP) from September 2001 – 2004 [8]. The highest incidence of CHARGE syndrome in Canada was estimated at 1:8,500 live births in provinces with a research interest in CHARGE syndrome [9]. The true incidence of CHARGE syndrome reported internationally may therefore be underestimated [10].

## Clinical description

CHARGE syndrome includes the following features:

### Coloboma

This feature may be unilateral or bilateral and may affect only the iris or extend to involve the retina, or only the retina. Vision may be normal or impaired. The eye abnormalities range from iris coloboma without visual impairment to microphthalmos and anophthalmos. Retinal coloboma is more prevalent than iris coloboma and can affect the optic nerve. Eye malformations have been reported in as many as 80% of patients with CHARGE syndrome, with retinal involvement being the most common [11]. External inspection is not sufficient and testing for functional vision is important but challenging especially in CHARGE individuals with extensive bilateral chorioretinal coloboma involving the optic nerve [12].

### Heart defect

Congenital heart defects occur in 75–80% of patients with CHARGE syndrome. The most common major heart defect is tetralogy of Fallot (33%). Other frequent anomalies are patent ductus arteriosus, double outlet right ventricle with atrioventricular canal, ventricular septal defect and atrial septal defect with or without cleft mitral valve. Vascular rings and more complex heart defects need to be anticipated [13-16].

### Atresia choanae

Choanal atresia is a narrowing or a blockage of the passages between the nasal cavity and the naso-pharynx. It represents a primary feature with a high index of suspicion for CHARGE syndrome and it should focus attention on other organ systems such as the eye and heart. Choanal atresia may be membranous or bony; bilateral or unilateral. Bilateral posterior choanal atresia (BPCA) was shown to be associated with increased neonatal mortality, especially if associated with major cardiac malformations +\- tracheoesophageal atresia [13]. However, the Canadian epidemiological study data suggests that an individual from this population with a more severe clinical presentation of CHARGE features generally survive [9]. Polyhydramnios in pregnancy is seen commonly in individuals with bilateral posterior choanal atresia, and may also be present without BPCA, probably due to an insufficient swallowing mechanism. Chronic middle ear infections and deafness can be associated complications of choanal atresia [17].

### Retardation of growth and development

Growth and developmental retardation become more obvious as the child matures. At birth, children with CHARGE syndrome usually have normal weights and lengths [18]. When growth deceleration is due to cardiac and respiratory problems, there may be catch up growth, and normal height can be obtained [19]. However, the influence of feeding problems on growth in infancy should not be underestimated. Early and continued intervention for feeding difficulties is vitally important [20]; occasionally there is growth hormone deficiency. The majority of school-aged children with CHARGE syndrome are below the third percentile for physical growth norms [18]; feeding with solids and lumpy foods, and risk of aspiration may still exist.

Mental retardation is variable with intelligence quotients (IQ) ranging from near-normal to profound retardation. Behavioral issues and an autism-like spectrum disorder are now being recognized as features of the syndrome [21,22].

The adult patient population is at risk of obesity (personal communication and observation by Dr. Kim Blake). Growth in height can occur in adults with CHARGE syndrome well into their 20's [19].

### Genitourinary problems

Under-development of the external genitalia is a common finding in males but it is more difficult to recognize in females. Microphallus, penile agenesis, hypospadias, chordee, cryptorchidism, bifid scrotum, atresia of uterus, cervix and vagina, hypoplastic labia and clitoris are reported genital anomalies in this syndrome. Reported renal anomalies include solitary kidney, hydronephrosis, renal hypoplasia, duplex kidneys and vesicoureteral reflux. Hypogonadotrophic hypogonadism has been reported and is associated with delays in puberty or pubertal arrest [23,24].

### Ear, olfactory and other cranial nerve anomalies

**Figure 1:**
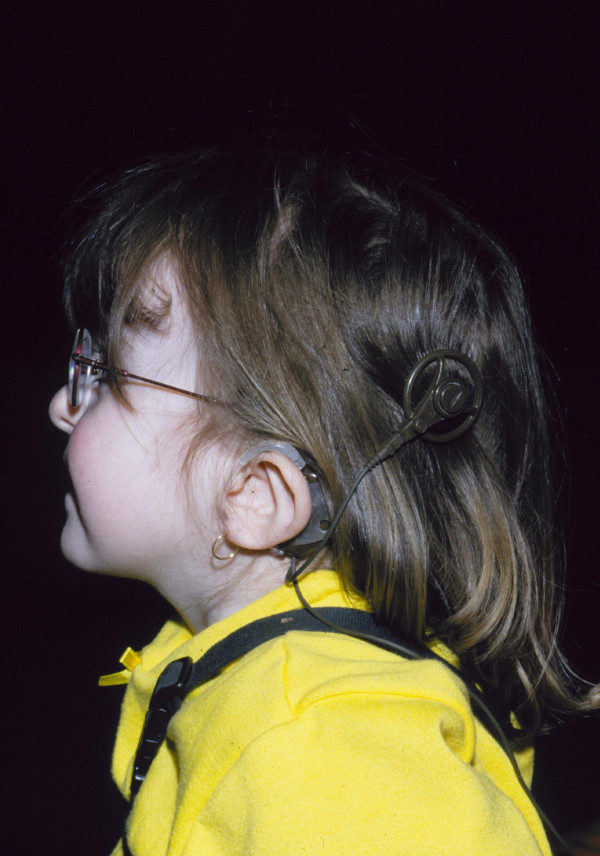
CHARGE syndrome: unusually shaped ears showing cochlear transplant.

Ear abnormalities include a classical finding of unusually shaped ears [6]. Lack of cartilage to the outer ear with deficient 7^th ^nerve innervation to intrinsic ear muscles produces a prominent lop- or cup-shaped ear with a hypoplastic lobule (Figure 1). Hearing loss, conductive and/or nerve deafness, ranges from mild to severe. Ear anomalies were reported in 80–100% of cases in different series [5,7,15,25]. Facial nerve palsies were noted to be a reliable predictor of sensorineural hearing loss. The characteristic abnormalities demonstrated by temporal bone computerized tomography (CT) or magnetic resonance imaging (MRI) scan include hypoplastic incus, decreased numbers of turns to the cochlea (Mondini defect), and, in particular, absent semicircular canals. These distinctive radiological findings are classical for CHARGE syndrome and can aid diagnosis in a suspected case [26]. For this reason, a neonatal CT scan to look at the choanae and temporal bones can be extremely useful.

The major diagnostic criteria for CHARGE syndrome [5] include cranial nerve (CN) anomalies, which are usually asymmetric. Cranial nerve dysfunctions include: CN I (anosmia). Absence or anomalies of the olfactory bulb are highly indicative of CHARGE syndrome [27] CN VII (facial palsy); CN IX/X/XI (swallowing problems, gastroesophal reflux, and velopharyngeal aspiration); and CN VIII (sensorineural hearing loss) [14,28]. CN V and CN II may also be involved [29].

### Behavioral phenotype

There are many challenging behaviors that are expressed in individuals with CHARGE syndrome. Multiple studies [21] from four different countries, using a variety of test instruments, came up with similar themes and similar behavioral patterns in children with CHARGE syndrome [21,22,30-34]. Children with CHARGE syndrome have relatively low adaptive behavior skills and motor impairments being particularly significant, with symptoms of autistic spectrum disorder (ASD). The behavior they display is often very adaptive to their environment and to their own disabilities. These behaviors may be partially related to problems with arousal and self-regulation. Taken together, the articles in this series reveal the emergence of behavioral phenotypes that are perhaps specific to CHARGE syndrome [21]. Data are emerging about the unique behavioral phenotype of CHARGE syndrome compared to other genetic syndromes such as Down syndrome, Prader-Willi syndrome and Williams syndrome [30] and the Autism spectrum.

## Etiology

There is a crucial stage of embryogenesis, when failure to rupture the primitive bucconasal membrane (35^th ^to 38^th ^day) brings about choanal atresia. Conotruncal cardiac defects can result from aberrations in cephalic neural crest cell migration during the 4^th ^and 5^th ^weeks after conception. The cochlear duct begins to develop around the 36^th ^day, and the eyes develop between days 34 and 44 days post-conception, which is also the time during which many cranial nerves are developing. All the malformations in CHARGE syndrome occur early during the first trimester. *In situ *hybridization analysis of the *CHD7 *gene during early human development showed a good correlation between *CHD7 *expression patterns and the developmental anomalies observed in CHARGE syndrome [35].

### Mutations in a member of the chromodomain gene family – CHD7

A team from Radboud University Nijmegen, The Netherlands, identified (by array-based comparative genomic hybridization) a small overlapping microdeletion at chromosome 8q12 in two patients with CHARGE syndrome. Within this region the candidate *CHD7 *gene was identified and sequenced in 17 patients. Initially, 10 patients had an identified mutation [36]. After improvement of the sequencing procedure, a mutation was found in 16 of the 17 original patients [7]. *CHD7 *is a large gene containing 38 exons. Most mutations found are stop or frame shift mutations resulting in truncation of the CHD7 protein. So far, no mutation hot spots have been found and mutations are scattered throughout the gene. In two large independent series of patients with CHARGE syndrome, mutations were found in 69 out of 107 (64%) CHARGE patients [7] and 64 of 110 (58%) CHARGE patients [25]. Both studies required full-gene sequencing of this large gene. Most mutations are found in patients who fulfill the clinical criteria for CHARGE syndrome, especially when they have absence of the semicircular canals and olfactory bulbs. The CHARGE phenotype may be related to the actual mutations within the *CHD7 *gene, as may the later onset features and behavioral phenotype. van Ravenswaaij found that a 26 year old woman with various CHARGE features and normal intelligence tested positive for the *CHD7 *mutation. Johnson [37] have also associated *CHD7 *mutations with CHARGE association by mapping a balanced chromosome translocation in affected monozygotic twins, thus confirming the earlier findings of the Dutch team. It is interesting to note that in the few reported studies of monozygotic twins with mutations in *CHD7*, there has been discordant expression of the syndrome, suggesting that genotype-phenotype predictions will remain imprecise.

## Diagnostic methods

### Genetic

• Karyotype to confirm the integrity of chromosome numbers 22, 14, and 9.

• Fluorescent *in situ *hybridization (FISH) to exclude 22q11 deletion.

• *CHD7 *gene mutation testing is now becoming available on a clinical basis. Molecular technologies are preferred because detection of deletions using FISH for the *CHD7 *gene is rare [36,38].

• Comparative genomic hybridization should be considered in patients with CHARGE syndrome and normal *CHD7 *mutation analysis results.

### Biochemical

Blood urea nitrogen, creatinine, electrolytes and calcium should be measured to evaluate renal function and exclude hypocalcemia. If hypocalcemia is found, T-cells should be evaluated for signs of DiGeorge sequence.

### Hormonal

• In cases of hypogenitalism, Luteinising Hormone Releasing Hormone (LHRH) and Human Chorionic Gonadotropin (HCG) tests should be performed to evaluate the pituitary gonadal axis (needs to be completed in the first four months of life or at puberty). Growth hormone (GH) stimulation levels should be investigated to exclude deficiency of GH as a cause for growth retardation.

• Frequent measurements of growth are required, prepubertally a left hand X-ray for bone age, followed by screening for hypogonadotrophic hypogonadism in early puberty.

### Cardiac

Echocardiogram to should be conducted to identify and/or exclude congenital cardiac defects. Holter monitoring should be used to detect rhythm abnormalities.

### Hearing

Audiometry and auditory brainstem response should be examined to document the type and severity of conductive and sensorineural hearing loss (often needs repeating, and different audiological tests may be performed as the child matures).

### Vision

Visual analysis, and electroretinogram and functional vision testing should be conducted to identify and document the severity of visual loss [11,12].

### Radiological

Skeletal survey should be carried out to exclude skeletal anomalies, particularly those of the cervical spine. Scoliosis is more prevalent than previously reported [41].

Frequent feeding assessments (depending on the severity of the feeding issues), including combinations of barium swallow, reflux scan, pH monitoring with a pediatric gastroenterologist and feeding team should be conducted to diagnose swallowing dysfunction, esophageal dysmotility, and frequent severe gastroesophageal reflux (GER) and tracheal aspiration [13,20].

Head CT and/or MRI scans, including imaging of the temporal bones should be performed to exclude cerebral malformation and defective formation of the ossicles of the middle ear, cochlear and semicircular canals. Detailed radiological visualization of the olfactory tracts and the cranial nerves are desirable and require fine cuts. The olfactory bulbs and tracts imaging may be pathognomonic for CHARGE syndrome [27].

Abdominal ultrasound and voiding cystourethrogram should be carried out to exclude renal anomalies.

Radiological testing should include a DEXA scan for osteoporosis.

## Differential diagnosis

Some characteristics of CHARGE syndrome overlap with those of other conditions including: VACTERL association, DiGeorge sequence [42], Velo-cardio-facial syndrome (VCFS), Cat Eye syndrome, retinoic acid embryopathy, and PAX2 abnormalities (The *PAX2 *gene is expressed in primitive cells of the kidney, ureter, eye, ear and central nervous system). Furthermore, several different structural chromosome abnormalities have been reported in children with coloboma, choanal atresia, and/or heart defects (some examples are: deletion 18q22.3-qter, duplication 2q37.3-qter, deletion 3p25.1-pter, deletion 22q11.1-qter, duplication 14q22-q24.3 and duplication 8q22-qter). Although still very rare, since the discovery of the *CHD7 *gene mutation, several patients with a submicroscopic deletion of 8q12 that includes the *CHD7 *gene have been reported [42].

It is important to rule out a submicroscopic chromosomal deletion of 22q11 with FISH analysis in all patients suspected of CHARGE syndrome, especially those with thymic hypoplasia and hypocalcemia. PAX2 abnormalities can lead to renal problems and ocular coloboma but few of the other features of CHARGE syndrome have been observed in such children [43].

The syndrome with hypoplasia of the depressor anguli oris muscle and cardiac defects also overlaps with both CHARGE syndrome and Velo-cardio-facial syndrome (VCFS). Retinoic acid embryopathy can produce ear, face, heart and cranial nerve defects similar to CHARGE syndrome, however, brain malformations resulting from retinoic acid embryopathy are usually much more severe. Exposure to retinoic acid during critical periods of morphogenesis has not been reported in children with CHARGE syndrome.

## Genetic counseling

Most cases of CHARGE syndrome are sporadic, occurring in an otherwise normal family. The presence of CHARGE syndrome like features should prompt a detailed evaluation of the family members including parents. There are a number of overlapping features with other conditions such as DiGeorge syndrome, VATER, Oculo-auriculo-vertebral syndrome thus all children/adults suspected with CHARGE syndrome should have a formal genetics consultation. The discovery of the *CHD7 *gene has led to the conclusion that most patients with CHARGE syndrome have a *de novo *autosomal dominant mutation. The first parent-to-child transmissions of *CHD7 *mutations, as well as germ line mosaicism, have already been identified by the Baylor group [25] and independently by the Vissers group [36]. The documented empiric recurrence risk is around 2%. Two separate studies have revealed that advanced paternal age is associated with sporadic cases of CHARGE syndrome [44,45]. The recent gene discovery will impact on genetic counseling and prenatal diagnosis issues.

## Antenatal diagnosis

Most of the abnormalities associated with CHARGE syndrome are difficult to diagnose antenatally through ultrasound unless there is a high index of suspicion with the presence of polyhydramnios. However, focused ultrasound for detection of external ear anomalies, choanal atresia, semicircular canal agenesis and arhinencephaly should lead to a higher prenatal defection rate. Moreover, arhinencephaly and semicircular canal agenesis were two constant features in fetuses with CHARGE syndrome and *CHD7 *mutations [35]. If the *CHD7 *mutation is discovered in the index CHARGE individual, then reliable prenatal diagnosis is possible by chorionic villus sampling or amniocentesis.

## Management

Children with CHARGE syndrome require intensive medical management as well as numerous surgical interventions. The most common neonatal emergencies in CHARGE syndrome involve cyanosis due to bilateral posterior choanal atresia and/or congenital heart defects, or the less common presentation of tracheoesophageal fistula. The primary foci of management should be airway stabilization and circulatory support [5].

All patients suspected of having CHARGE syndrome should have a cardiology consultation. If the infant has restrictive pulmonary blood flow and is dependant on a patent ductus arteriosus, the administration of prostaglandin to maintain ductal patency may be life saving. Some children require tracheostomy to manage chronic airway problems and/or gastroesophageal reflux and aspiration. Children with CHARGE syndrome require aggressive medical management of their feeding difficulties, often needing gastrostomy and jejunostomy feeding tubes. Gastrooesophageal fundoplication may be required for GER that does not respond to medical management. As intubation can be difficult in children with CHARGE syndrome, a pediatric anesthesiologist or pediatric otolaryngologist should be present for planned surgical procedures.

Any infant suspected of having CHARGE syndrome should have a complete eye examination by an ophthalmologist, with follow-up every three to six months thereafter, depending on the eye involvement. Photophobia is often a significant problem that can be ameliorated with tinted spectacles or by wearing a cap or visor with a dark brim. In the presence of facial palsy, patients should avoid corneal scarring by using artificial tears.

Hearing aids should be used as soon as hearing loss is documented. Frequent re-molding of the earpieces is necessary as the ear canals can be initially very small and ear cartilage may be insufficient to support a hearing aid. Cochlear implantations have been successfully performed in CHARGE syndrome patients. Children with CHARGE syndrome who undergo cochlear implantation should be allowed to continue with their sign language in parallel with their expressive speech training [46]. Adapted educational and therapeutic services to deal with dual auditory and visual sensory impairment should be proposed early in the child's life [44-46]. However, this population is unique with respect to their aberrant cranial nerve pathways and problems with expressive language.

In terms of endocrine issues, sex steroid therapy has been used for penile growth and descent of testes in males with CHARGE syndrome. The main use for testosterone is for delayed and incomplete male puberty during adolescence. Females often require hormone replacement at puberty [23]. Sex hormone replacement is also indicated for prevention of osteoporosis [24].

## Unresolved questions

"Will early recognition and treatment of infants with CHARGE syndrome improve their clinical and behavioral well being?"

Clearly, early detection of the medical issues will have an impact on the ultimate prognosis. Even so, the number of medical issues in CHARGE syndrome make this population very vulnerable to different clinical problems. Similarly, the neurological outcome will depend on the promptness of medical care and support services and any underlying cranial anomalies.

"Is there a genotype/phenotype correlation between the different CHARGE features and the different mutations in the *CHD7 *gene?"

In a study of 110 patients [25] it has been shown that cardiovascular malformations, coloboma and facial asymmetry are common findings in CHARGE syndrome caused by *CHD7 *mutation. Further studies of this kind will increase understanding of the genotype/phenotype correlation in CHARGE syndrome.

"Is there a distinct behavioral phenotype for CHARGE syndrome and does this correlate with the different *CHD7 *mutations?"

We are seeing certain patterns of behavior such as obsessive compulsive and in the pervasive spectrum (autism spectrum disorders).
